# Skin and Soft Tissue Lesions in a District Hospital in Central Nigeria: A Histopathological Study

**DOI:** 10.1155/2019/8143680

**Published:** 2019-12-26

**Authors:** Babatunde M. Duduyemi, Abidemi E. Omonisi, Nicholas A. Titiloye

**Affiliations:** ^1^Department of Pathology, Kwame Nkrumah University of Science and Technology, Kumasi, Ghana; ^2^Department of Pathology, Asokoro District Hospital, Abuja, Nigeria; ^3^Department of Anatomic Pathology, Ekiti State University, Ado Ekiti, Nigeria

## Abstract

*Introduction*. Skin and soft tissue diseases form a large and heterogeneous group of mesenchymal extraskeletal and dermatologic lesions in humans. Diseases of the skin and soft tissue can develop virtually anywhere in the body, extremities, the trunk, the retroperitoneum, the head, and the neck. Our study aims to review skin and soft tissue specimens from our centre describing the histopathological patterns.

**Method:**

A cross sectional study was done using secondary data of all skin and soft tissue specimens over a 3 year period. Patients' demographics, sites of specimen, and histological diagnoses were extracted from the surgical day book. The data were analysed in terms of age and sex distribution and histological characteristics of pathologic lesions using the SPSS version 22. The data for these patients were presented in tables and figures.

**Result:**

451 skin and soft tissue specimens constituting 18% of all the specimens with an M : F ratio of 1 : 1.2. The age range of our patients was 4–85 years with a mean of 33.52 ± 15.05 years. The peak age of occurrence was 30–39 years. Most of our cases were seen in the extremities (50.7%) followed by head (22.2%), while the least common sites were the perineal and neck areas (5.3% each). The commonest site in females was the upper limb (32.4%); the head and lower limb were the commonest sites in males (28.4% each). Most of our patients have neoplastic lesions of skin and soft tissue constituting 68.3%, inflammatory lesions (16.9%), and the least common lesion being hamartoma (0.2%). The most common category of lesions includes inflammatory (nonspecific dermatitis 6.5%); cysts (dermoid cyst 6%); reactive (hypertrophic scar 1%); and neoplastic (lipoma 32.4%). The benign neoplasms were more common (92.9%) than the malignant ones (7.1%). The neoplastic lesions were relatively more common in males than females and the reverse was true for the inflammatory lesions.

**Conclusion:**

Skin and soft tissue lesions are relatively common in our environment with majority being benign neoplastic lesion.

## 1. Introduction

Patients in Sub-Saharan Africa commonly present to the physicians with skin and soft tissue lesions, either as the chief compliant or as a manifestation of an underlying illness.

Skin and soft tissues diseases form a large and heterogeneous group of mesenchymal extraskeletal and dermatologic lesions in humans [1–3]. Diseases of the skin and soft tissue can develop virtually anywhere in the body, extremities, the trunk, the retroperitoneum, the head, and the neck [4].

The scope of lesions seen in skin and soft tissue may be categorized as congenital, acquired, infective lesions, benign, and malignant lesions [5–10]. The exact aetiology of skin and soft tissue lesions is unknown. However, predisposing factors such as eruption of multiple lesions following chemical exposure such as sulphur mustard gas and glycol ether solvent (2-butoxyethanol), hormones (especially during pregnancy and involution of the uterus), rearrangements of chromosomes, and trauma have been documented in the English literature [11–15].

Several assorted histological types of skin and soft tissue lesions have been reported across nations of the world [16–20]. However, the management of skin and soft tissue diseases in low- and middle-income countries is faced with many challenges ranging from late presentation to health facilities and limited financial resources provided by Government to settlement of hospital bills by patients from their personal resources and difficulty in establishing the correct diagnosis due to paucity of experts in these regions who can satisfactorily handle skin and soft tissue diseases [21, 22]. Nonexistence of ancillary diagnostic techniques such as immunohistochemistry services and fluorescence microscopy which are very relevant in ascertaining correct pathologic diagnosis is also lacking [23, 24].

The main objective of this study is to share our experience with skin and soft tissue lesions from a district health centre in an urban city in Sub-Saharan Africa by describing the histopathological patterns of 423 consecutive cases.

## 2. Methodology

A cross-sectional study using secondary data was undertaken to review the histopathology reports of all skin and soft tissue specimens submitted at the Department of Pathology of District Hospital, in Central Nigeria, from November 2009 to November 2012. Our laboratory is a referral centre for 12 other government district hospitals and private hospitals in the Federal Capital Territory and its environs. Patients' demographics, sites of specimen, and histological diagnoses were extracted from the surgical day book. The slides were retrieved and reviewed for confirmation of diagnoses, and those not available had their blocks cut fresh and stained with H&E for review. The data were analysed in terms of frequency, age and sex distribution, as well as histological characteristics of pathologic lesions using the SPSS version 22. The data for these patients were presented in tables and figures.

## 3. Results

A total of 451 skin and soft tissue specimens were received over the 3-year period constituting 18% of all the specimens. There were 201 males and 250 females with an M : F ratio of 1 : 1.2. The age range of our patients was 4–85 years with a mean of 33.52 ± 15.05 years.

### 3.1. Age and Sex Distribution

The age and sex distribution of the patients is shown in Table 1. The peak age of occurrence in this study is 30–39 years followed by 40–49 and 20–29 age groups; all constituting about 70% of the cases. The least number of cases were seen after the age 70 years constituting 1.3%.

### 3.2. Frequency Distribution of the Lesions with Site

**Figure 1:**
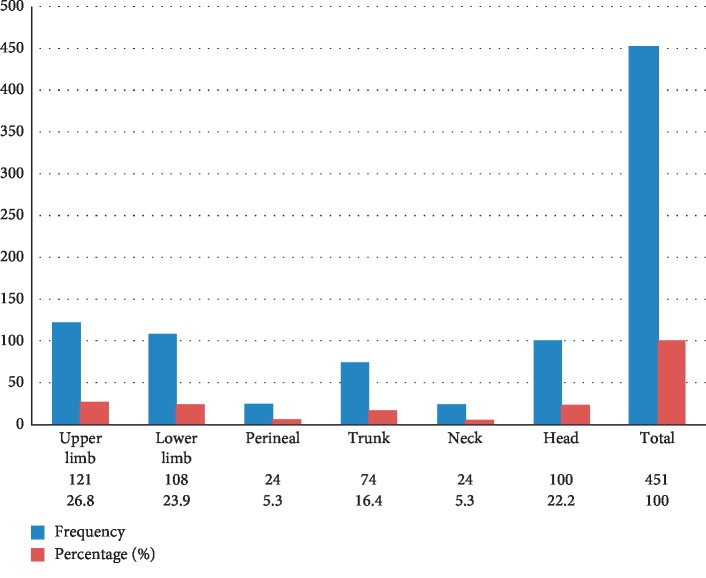
Frequency distribution of the lesions with site.

This is as shown in Figure 1. Most of our cases were seen in the upper and lower limbs constituting 50.7% followed by head region (22.2%), while the least common sites are the perineal and neck areas constituting 5.3% each.  Table 2 shows the relationship of sites with gender. While the most common site in females was the upper limb (32.4%), the head and lower limb are the most common sites in males constituting 28.4% each (Table 3).

### 3.3. Histologic Diagnosis

**Figure 2:**
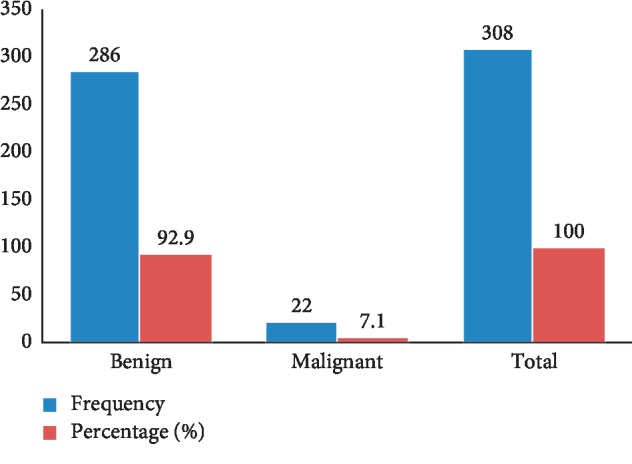
Frequency distribution of neoplastic lesions in our patients.

The distribution of the histologic diagnoses is as shown in Table 4. Most of our patients have neoplastic lesions of skin and soft tissue constituting 68.3%; followed by inflammatory lesions (16.9%) and the least common lesion being growth disorder (hamartoma (0.2%)). The most common category of lesions includes inflammatory (nonspecific dermatitis 6.5%); cysts (dermoid cyst 6%); reactive (hypertrophic scar 1%); and neoplastic (lipoma 32.4%). The benign neoplasms were more common and constitute 92.9%, while the malignant ones constitute 7.1% as shown in Figure 2. The neoplastic lesions were relatively more common in females than males, and reverse was true for the inflammatory lesions. The only growth disorder (hamartoma) occurred in a female. The distribution of benign and malignant neoplasms is shown in Table 5. The most common lesion in our study is lipoma (*n* = 146/451) constituting about 47% of all neoplastic cases and 51.7% of benign neoplasm. Basal cell and squamous cell carcinomas were the two most common malignant neoplasms constituting 22.73% each.

## 4. Discussion

Until today, skin and soft tissue lesions are often neglected in our part of the world unless they are associated with severe pains and life-threatening conditions. Most patients with surgical problems that are routinely treatable in high-income countries never reach a health facility or are treated at a facility with inadequate human or physical resources [25].

In Sub-Saharan Africa, majority of these surgical problems are skin and soft tissue- related diseases, many of which will never reach the health facilities with consequent low volume of surgical materials received in most histopathology laboratories in Nigeria and other parts of Sub-Saharan Africa. However, the story is changing due to number of factors, chief among these factors are the increasing awareness of cosmetics especially among the females and the increasing availability of the numbers of competent specialists capable of effectively managing patients with skin and soft tissue lesions in this region.

This study is the first to describe the pattern of skin and soft tissue lesions in this District Hospital in Central Nigeria. Although this study was limited to a single hospital, we believe that our findings could be used to project accurately for the histopathological pattern of skin and soft tissue lesions in the entire North Central region of Nigeria.

In this series, more cases were seen in the females when compared with males. This finding is similar to reports by Kumar et al. [26] who reported preponderance in the females than the males but contradicts the findings by Ki and Rotstein who reported a higher incidence among males [27]. The possible reason is that more cases were seen in the females in this study which may be connected to the fact that females are more readily accessible to health facilities for proper treatment of ailments than the males, who are always very busy in the capital city where this study was conducted and hardly visit the hospitals unless they are in severe pain or during emergency.

We discovered from this study, the age group 30–39 years presented with the highest frequency of skin and soft tissue lesions with 29.9%. This was distantly followed by age group 40–49 with 20.2%. Abubakar et al. [28] from the same setting reported their highest frequency among age group 20–29. We could conclude that skin and soft tissue lesions are commoner among the reproductive group age in our environment.

In this current study, the topographic distribution of skin and soft tissue lesions was categorized into six main sites in the decreasing order of frequency of involvement which were the upper limb, lower limb, head, trunk, and neck. However, majority of the lesions affected both extremities when combined compared with other sites. This finding is similar to what was recorded by Miettinen [29] who reported that leiomyosarcomas can occur at any site, although they were more frequent in the retroperitoneum and proximal extremities. Morrison [30] also reported that over 50% of soft tissue sarcoma most commonly occurs in the extremities.

Histopathologically, the skin and soft tissue lesions were further broadly classified into growth disorder, reactive, cystic, inflammatory, and neoplastic accounting for 0.2%, 1.6%, 13.1%, 16.9%, and 68.3%, respectively. The neoplastic lesions were further subdivided into benign and malignant lesions. The benign lesions accounted for vast majority of the neoplastic lesions representing 92.9% of the cases, while the malignant lesions accounted for only 7.1%. In a study by Kirby et al., among 83 patients who had a soft tissue tumour in the foot, 72 (87%) of the lesions were benign, while the remaining 11 (13%) were malignant [31]. This report was compatible with our finding in this study, although we reported a higher percentage of the lesions as benign and lower percentage as malignant. The bottom-line was that the benign lesions constituted the vast majority of the cases in both studies.

Unexpectedly, no potentially malignant or premalignant lesion was recorded in this study. This observation was in agreement with the findings of Sharma et al. that reviewed the histopathological patterns of head and neck lesions of 145 cases. They also observed that no premalignant or potentially malignant lesions were recorded in their series [32]. The most common benign neoplasm in our study is lipoma, constituting about half of the cases. This is similar to the findings in most studies of benign skin and soft tissue neoplasms. Lipoma is more common in females than males which agrees with most studies [14, 17, 20]. Squamous papilloma is the second most common benign neoplasm in our study. Abubakar et al. [32] in Nigeria found the same to be true in their study on the histopathology of the skin. The most common malignant neoplasms are squamous cell and basal cell carcinoma closely followed by Kaposi sarcoma. The studies of Abubakar et al. (Northern Nigeria) and Oseni et al. [28, 33] (Western Nigeria) found melanoma to be the most common, while Ochicha et al. [34] and Ahachi et al. [35] (Northern Nigeria) reported squamous cell carcinoma to be the most common. All the cases of Kaposi sarcoma in our study were associated with HIV which is keeping with finding in literature [33–35].

The diagnosis and treatment of skin and soft tissue lesions continue to present with challenges because lesions related to these systems of the body are more often diversified and each disease entity has its own distinctive anatomic, epidemiologic, natural history, clinicopathological features, and treatment considerations.

The diagnoses of skin and soft tissue lesions by the pathologists are usually supported by adequate clinical history and clinical assessment, ancillary investigations and tissue-based diagnosis which are ultimate in making correct diagnosis. Therefore, the correct diagnosis and management of patients with skin and soft tissue diseases will require teamwork, skills, clinicopathology correlation of cases, and interdisciplinary collaborations. Development of practical guidelines and minimum datasets for skin and soft tissue diseases will definitely help in the easy recognition cases and promote uniformity and comparison of cases between regions, thereby promoting collaborative research works on skin and soft tissue lesions.

Finally, this study has highlighted the significance of prompt diagnosis and interdisciplinary clinicopathology correlation as vital links in the management of skin and soft tissue lesions particularly in Sub-Saharan Africa where such practice is not prevalent.

## 5. Conclusion

Our study showed that skin and soft tissue lesions are very common in our environment ranging from neoplastic to inflammatory and cystic to reactive changes. The benign neoplasms are the most common lesions which compared favourably with other studies in Africa and the developed world.

## Figures and Tables

**Table 1:** General characteristics of our patients by gender and age.

Ages	Gender (%)		
	Male	Female	Total
0–9	13 (6.5)	19 (7.6)	32 (7.1)
10–19	24 (11.9)	26 (10.4)	50 (11.1)
20–29	31 (15.4)	55 (22.0)	86 (19.1)
30–39	54 (26.9)	81 (32.4)	135 (29.9)
40–49	47 (23.4)	44 (17.6)	91 (20.2)
50–59	23 (11.4)	14 (5.6)	37 (8.2)
60–69	6 (3.0)	8 (3.2)	14 (3.1)
70–79	1 (0.5)	1 (0.4)	2 (0.4)
80–89	2 (1.0)	2 (0.8)	4 (0.9)
Total	201	250	451 (100)

**Table 2:** Relationship between the gender and site of the lesion.

Gender	Sites (%)						
	Upper limb	Lower limb	Perineal	Trunk	Neck	Head	Total, *n* (%)
Male	40 (19.9)	57 (28.4)	9 (4.5)	31 (15.9)	6 (3.0)	57 (28.4)	201 (44.6)
Female	81 (32.4)	51 (20.4)	15 (6.0)	42 (16.8)	18 (7.2)	43 (17.2)	250 (53.4)
Total	121 (26.8)	108 (23.9)	24 (5.3)	74 (16.4)	24 (5.3)	100 (22.2)	451 (100)

**Table 3:** Relationship between the ages and site of the lesion.

Ages	Sites (%)							
	Upper limb	Lower limb	Perinea l	Trunk	Neck	Head	Total	*P* value
0–9	9 (28.1)	8 (25.0)	2 (6.2)	1 (3.1)	6 (18.8)	6 (18.8)	32 (100)	0.346
10–19	12 (24.0)	11 (22.0)	2 (4.0)	9 (18.0)	2 (4.0)	14 (28.0)	50 (100)	
20–29	18 (20.9)	22 (25.6)	5 (5.8)	13 (15.1)	2 (2.3)	26 (30.2)	86 (100)	
30–39	41 (30.4)	25 (18.5)	12 (8.9)	25 (18.5)	7 (5.2)	25 (18.5)	135 (100)	
40–49	26 (28.6)	27 (29.7)	2 (2.2)	17 (18.7)	4 (4.4)	15 (16.5)	91 (100)	
50–59	10 (27.0)	11 (29.7)	1 (2.7)	6 (16.2)	1 (2.7)	8 (21.6)	37 (100)	
60–69	4 (28.6)	3 (21.4)	0 (0.0)	2 (14.3)	2 (14.3)	3 (21.4)	14 (100)	
70–79	0 (0.0)	0 (0.0)	0 (0.0)	1 (50.0)	0 (50.0)	1 (50.0)	2 (100)	
80–89	1 (25.0)	1 (25.0)	0 (0.0)	0 (0.0)	0 (0.0)	2 (50.0)	4 (100)	
Total	121 (26.8)	108 (23.9)	24 (5.3)	74 (16.4)	24 (5.3)	100 (22.2)	451 (100)	

**Table 4:** Distribution and comparison of types of lesions with gender.

Type of lesion	Male	Female	Frequency	Percentage (%)
Neoplastic	132 (65.7)	176 (70.4)	308	68.3
Inflammatory	40 (19.9)	36 (14.4)	76	16.9
Cyst	26 (12.9)	33 (13.2)	59	13.1
Reactive	3 (1.5)	4 (1.6)	7	1.6
Growth disorder	0 (0.0)	1 (0.4)	1	0.2
Total	201 (100)	250 (100)	451	100

**Table 5:** Frequency distribution of benign and malignant neoplasms.

Benign				Malignant			
Types	Male	Female	Total	Types	Male	Female	Total
Lipoma	51 (17.48%)	95 (33.57%)	**146 (51.05%)**	Embryonal rhabdomyosarcoma	1 (4.55%)	0 (0%)	**1 (4.55%)**
Lymphagioma	3 (1.05%)	0 (0%)	**3 (1.05%)**	Peripheral nerve sheath tumour	1 (4.55%)	0 (0%)	**1 (4.55%)**
Dermatofibroma	4 (1.39%)	11 (3.85%)	**15 (5.25%)**	Dermatofibrosarcoma			
Protuberous	1 (4.55%)	1 (4.55%)	**2 (9.10%)**				
Neurofibroma	6 (2.10%)	7 (2.45%)	**13 (4.55%)**	Fibrosarcoma	1 (4.55%)	0 (0%)	**1 (4.55%)**
Nevus	3 (1.05%)	6 (2.10%)	**9 (3.15%)**	Kaposi sarcoma	1 (4.55%)	2 (9.10%)	**3 (13.65%)**
Squamous papilloma	10 (3.50%)	18 (6.29%)	**28 (9.79%)**	Squamous cell carcinoma	2 (9.10%)	3 (13.64%)	**5 (22.73%)**
Capillary haemangioma	15 (5.24%)	10 (3.50%)	**25 (8.74%)**	Basal cell carcinoma	2 (9.10%)	3 (13.64%)	**5 (22.73%)**
Nodular tenosynovitis	5 (1.75%)	7 (2.45%)	**12 (4.10%)**	Osteosarcoma	1 (4.55%)	0 (0%)	**1 (4.55%)**
Verruca vulgaris	14 (4.90%)	8 (2.80%)	**22 (7.70%)**	Melanoma	1 (4.55%)	1 (4.55%)	**2 (9.10%)**
Oncocytoma	1 (0.35%)	0 (0%)	**1 (0.35%)**	Pleomorphic sarcoma	1 (4.55%)	0 (0%)	**1 (4.55%)**
Eccrine spiradenoma	3 (1.05%)	3 (1.05%)	**6 (2.1%)**	**Total**	**12 (54.55%)**	**10 (45.45%)**	**22 (100.00%)**
Desmoid tumour	1 (0.35%)	0 (0%)	**1 (0.35%)**				
Schwannoma	4 (1.49%)	1 (0.35%)	**5 (1.75%)**				
**Total**	**120 (41.96%)**	**166 (58.04%)**	**286 (100.00%)**				

## Data Availability

The excel data used to support the findings of this study may be released upon application to the Committee on Human Research, Publication and Ethics of Asokoro District Hospital, Abuja Nigeria.
